# Statistical Inquiry into the Present State of the Medical Charities in Ireland, &c.

**Published:** 1836-01-01

**Authors:** 


### STATISTICAL INQUIRY INTO THE PRESENT STATE OF THE MEDICAL CHARITIES IN IRELAND, &c. By *Denis Phelan*, Surg. Dublin, 8vo, pp. 325.

THE œconomy of the Irish medical charities is very different from that of similar institutions on this side of the Channel. Like most of the French Hospitals and Maisons de Santé, they are supported chiefly at the expense of the Government; and the internal management of their affairs, and, therefore, the patronage connected with the greater number of them, are almost entirely in the hands of official men. This is a state of things which is ever to be regretted; for, while this exists, the works of charity are not the offspring of that tender and amiable feeling which delights to do active good without display, but are the mere ordinances of public authorities, which endeavour to mitigate one evil, under the dread of incurring another of greater magnitude. The annual expense of the Irish medical charities amounts to upwards £163,000., and it appears that this sum is derived from the following sources :—

| | |
|---|---|
| Treasury Grants to County Infirmaries .......... | £ 2,653 |
| Annual Parliamentary Grants to Dublin Hospitals.. | 14,374 |
| County Presentments.......................... | 82,839 |
| Private Subscriptions and Donations ............ | 39,078 |
| Petty Sessions and other Fines.................. | 1,742 |
| Produce of Property belonging to several Hospitals.. | 23,225 |
| | £163,911 |

By the 5th and 6th Geo. III. it is provided, that one infirmary for the relief of the diseased poor shall be erected and established in each county in Ireland. The funds of these county infirmaries are to be derived partly from county presentments (£600. per ann. granted by the grand juries), petty-sessions fines, treasury grants, and private donations and subscriptions; and the salary of the medical officer (£200. per ann. provided there is only one infirmary in the county) is paid, one half from the public treasury, and the other half from a county fine, levied by the grand jury. The proportion which the amount of the private funds of these county hospitals bears to that of the public funds is very small; affording thus the most satisfactory proof, that the respectable inhabitants of the districts feel but little interest, and exercise but little control, on the affairs of these charities. We may take the year 1828, as affording a fair estimate of the state of their funds. It appears, from the official report of the Board of Health for that year, that the whole amount of donations, subscriptions, petty-sessions fines, and other contingencies, was only £1973. for twenty-nine counties, whilst the treasury grants and county presentments were no less than £17,022.

The governors of these county infirmaries consist of the Primate, the Lord Chancellor, the Bishop of the diocese, and the Rector or Vicar of the parish, in which the infirmary is built, along with donors of twenty guineas, and annual subscribers of three guineas.

Mr. Phelan has pointed out, at great length, the principal defects of these Irish county infirmaries, and we trust that the members of the legislature, and all who feel an immediate interest in the well-being of Ireland, will attentively consider the objections which he has urged. The great medical blemishes in the organization of these establishments are the two following :—The eligibility of the surgeons and physicians being limited exclusively to graduates of the Dublin medical and surgical colleges ; and, secondly, the appointment, at least in many cases, of only one surgeon or physician to an infirmary.

In reference to the first of these blemishes, we are sure that we express the firm conviction of every liberal medical man, that such a state of things ought not to be tolerated any longer. We can suppose that the enlightened councils of the medical and surgical colleges in Dublin, may be actuated by a similar spirit to that which has so long *distinguished* the same fraternities in this metropolis; and that they wish us to believe, that no physician or surgeon can exercise his profession properly in Ireland, on the one hand, and in England on the other, unless he has derived his license from their respective boards; but we trust that a new and a better æra is about to commence speedily, and that all local and exclusive privileges of medical corporations will be utterly swept away. To argue the question of such a monopoly, as it was capable of dispute, ought always to have been, and thanks to Mr. Warburton now is, quite unnecessary. The simple position, that a medical man who is deemed fit, by any publicly-recognized university or college, to practise his profession in one part of the kingdom, ought surely to be capable of doing it equally well in all others, is a truism so obvious, and, withal, so reasonable, that we shall ever regard it as one of the foulest blots in the present existing corporations, that they have striven hard on all occasions, and by all means, to oppose and to nullify its spirit and operation. We now pass on to the other defect in the medical constitution of the Irish county hospitals—that, namely, of many of them having but one physician or surgeon, and having no competent medical officer residing within their walls. In some of the infirmaries, indeed, the surgeon himself resides in part of the building, and acts as apothecary at the same time. Mr. P. very properly does not approve of this arrangement, on the ground that no able or ambitious medical man would accept of the appointment if he was obliged to reside within the hospital, and was debarred, at the same time, from engaging in private practice. But, while he deprecates such an arrangement, he very justly condemns the appointment of one medical officer only to any infirmary, however small, as at once most unfair to the other professional men living in the neighbourhood, and most injurious, in many respects, to the patients admitted. Mr. P. might extend his reprobation to a multitude of other medical institutions, as well as to his own Irish county infirmaries. Some of the London hospitals are far from being efficiently conducted; and we believe that one of the main causes of this inefficiency, is the circumstance of the medical officers being too few in proportion to the multitude of in-door, as well as of out-door patients. Infinite good would result from the application of Adam Smith's principle of subdivision of labour, to some of the metropolitan hospitals. The suffering poor, as well as the body of the profession, would be greatly benefited by the change. Of course there must be a limit to the numbers of the medical officers of hospitals. Yet, on every account there should be as many as there can be consistently with the proper performance of duties adequately defined.

In addition to the regular infirmaries of each county in Ireland, there are numerous fever hospitals and general dispensaries, all more or less supported by public funds, and not dependent solely, as is the case throughout England and Scotland, on voluntary contributions.

Perhaps no fact can more loudly proclaim the unhappy state of society in our sister country, than that almost all her charities, for the relief of the poor sick, are to a certain degree compulsory. We do not mean by this to condemn, but only to lament the system. It is no doubt necessary, considering the manifold evils which afflict the country—the absence of poor laws, the absenteeism of the wealthy landlords and proprietors, the wretched fare and miserable shelter of the peasantry and working classes, and the consequent appalling prevalence of fevers and other endemic diseases. Mr.

Phelan estimates the annual amount of fever cases in Ireland to be not less than 108,000, or one in seventy-two of the whole population. By various recent Acts of Parliament, the Grand Juries are authorised "to present to fever hospitals, in any county, city, or town, any sum not exceeding double the amount raised by donation or subscription, and actually received by the treasurer; and, to dispensaries, any sums equal in amount to those sworn to as having been received by the treasurers of these institutions."

Mr. P. very feelingly adverts to the distressing insufficiency of fever hospitals (about sixty-eight in number) to afford adequate relief to the annually recurring wide-spread mischief. The number of dispensaries, receiving public support from county presentments, &c. exceeds 400. The salaries of the medical officers attached to these institutions vary, from 50*l.* to 120*l.* per annum; but, unfortunately and unfairly, the amount of remuneration is by no means, in all cases, proportionate to the amount of labour and fatigue incurred. Some of the surgeons are not resident in their districts, being five and six miles distant from them, and visiting them only twice or thrice a week; and we are assured by Mr. Phelan, that the most unworthy feelings of favouritism, political and religious partizanship, &c. are too often permitted to influence the governors of these charities, in their selection of the medical officers. We must refer our readers to his work for instances (and some of them are, indeed, most flagrant) in point. Like all other institutions, dispensaries, and, it would seem, the Irish ones in particular, are subject to shameful abuses; for what was intended to be a refuge only to the helpless and indigent, is too often used by those who are quite able to pay for professional assistance as a means of obtaining professional relief, without the shame of begging for it. Perhaps medical men have partly themselves to blame; they are always too ready to proffer their advice, and to devote their time and exertions, without an adequate remuneration.

The late exposures of the dirty trafficking of some country doctors, to obtain the parochial practice, under the Poor-Law-amendment Bill, at the dignified rate of half-a-crown a case, are melancholy comments on the state of the medical profession in this country.

To return to Mr. Phelan, we have the sincerest pleasure in strongly recommending his work to the attentive notice of the Irish public. He has shewn himself a most zealous and disinterested philanthropist; and we trust that his advice and co-operation will be called into requisition, by those who truly wish to mitigate the distresses of his country.

